# Long noncoding RNA DLGAP1-AS2 facilitates Wnt1 transcription through physically interacting with Six3 and drives the malignancy of gastric cancer

**DOI:** 10.1038/s41420-021-00649-z

**Published:** 2021-09-20

**Authors:** Jiawei Lu, Ying Xu, Wenjie Xie, Yinbing Tang, Heteng Zhang, Beibei Wang, Ji Mao, Tao Rui, Pengcheng Jiang, Wenbo Zhang

**Affiliations:** 1 Department of General Surgery, Affiliated People’s Hospital of Jiangsu University, Zhenjiang, China; 2 Department of Laboratory Center, Affiliated People’s Hospital of Jiangsu University, Zhenjiang, China; 3 Medical School of Nantong University, Nantong, China; 4 Department of Medicine, Affiliated People’s Hospital of Jiangsu University, Zhenjiang, China

**Keywords:** Cancer, Gastrointestinal cancer

## Abstract

The long noncoding RNA (lncRNA) DLGAP1-AS2 has recently been characterized as an oncogenic lncRNA in several cancers. However, its biological roles and clinical significance in gastric cancer (GC) remains barely understood. In this study, we performed a systematic analysis of DLGAP1-AS2 expression with data from the TCGA and GEO database as well as our clinic GC samples. In agreement with previous studies, our findings demonstrated that DLGAP1-AS2 was significantly up-regulated in GC and its high expression was associated with poor prognosis, suggesting that DLGAP1-AS2 might be a putative oncogenic lncRNA of GC. Loss of DLGAP1-AS2 restricted cell proliferation, migration, and invasion in GC cell lines. Mechanically, Wnt1 was identified as the downstream target of DLGAP1-AS2 by using bioinformatics analysis coupled with qPCR and Western blot assays. Furthermore, DLGAP1-AS2 was found to directly interact with the transcriptional repressor Six3, and this interaction hampered Six3 binding to the promoter regions of the Wnt1 gene, thereby leading to transcriptional activation of Wnt1. Consequently, GC cells lacking DLGAP1-AS2 showed a decreased Wnt1 expression and weakened Wnt/β-catenin signaling. Further, Six3 silencing could reverse the above effects, highlighting a pivotal role of Six3 in the DLGAP1-AS2-mediated activation of Wnt/β-catenin signaling. Either genetic (Wnt1 knockdown) or pharmacological (LF3) inhibition of Wnt/β-catenin signaling could effectively abolish the activation of Wnt/β-catenin signaling by Six3 depletion, thereby preventing GC cell malignant transformation. Taken together, our results suggest that DLGAP1-AS2 functions as an oncogenic factor by directly interacting with Six3 to relieve its suppression on Wnt1 expression, thereby driving the malignancy of GC. DLGAP1-AS2/Six3/Wnt1/β-catenin signaling axis might serve as a promising diagnostic and therapeutic target for GC.

## Introduction

Gastric cancer (GC) is a growing public health concern worldwide, ranked as the fifth most common malignant tumor and the third most common cause of cancer death globally [1]. China Cancer Data Report in 2015 shows that the estimated number of new cases of GC in China was 679,000, and the number of deaths was 498,000 [2]. Such high morbidity and mortality are not surprising because the initial signs and symptoms of GC are not obvious and the diagnosis rate of early GC is relatively low [3]. To date, some serum tumor biomarkers, including carcinoembryonic antigen (CEA), cancer antigen 19-9 (CA19-9), and CA72-4, have been used in diagnosing GC in asymptomatic patients but the results are unsatisfied due to low sensitivity and low positive predictive value [4]. Therefore it is vital to discover effective biomarkers and targets for GC diagnosis and treatment.

Wnt signaling is one of the most important signaling pathways in regulating development and cancer. It is recognized that aberrant activation of the Wnt signaling pathway promotes GC development, which can be found in more than 30% of GC patients [5]. Generally, the Wnt signaling pathway is classified into two categories: canonical Wnt/β-catenin (Wnt/β-catenin dependent pathway) and the non-canonical Wnt/β-catenin pathway (β-catenin independent pathway) [6]. The canonical Wnt/β-catenin pathway regulates the stabilization and the shuttle of β-catenin to the nucleus and subsequently regulates target gene transcription [7]. In the absence of the Wnt ligand, cytoplasmic β-catenin is degraded by the destruction complex including the AXIN, APC, and GSK-3β proteins [8]. In the presence of the Wnt ligand, however, it interacts with its receptor complex consisting of low-density lipoprotein receptor-related protein 5/6 (LRP5/6) and Frizzled (FZD), and leads to the activation of the Wnt/β-catenin pathway [9]. And then, cytoplasmic protein Dvl/Disheveled (Dsh) is recruited to the cell membrane and activated by phosphorylation, and therefore promoting β-catenin translocation to the nucleus, where it interacts with coregulators of transcription, including T cell factor/lymphocyte enhancer factor (TCF/LEF) to form a β-catenin/TCF/LEF complex. The transcriptional activation of β-catenin downstream targets, including MMP-9, Myc, Axin2, and cyclin D1, is able to promote cell cycle progression, cell proliferation, and metastasis in the progression of cancer [10–12].

The transcription factor Six3 is a member of the SIX family and is generally regarded as a tumor suppressor. Six3 has been previously reported to be a negative regulator of the Wnt pathway in several cancers [13]. In nonsmall cell lung cancer (NSCLC), Six3 inhibits NSCLC cell proliferation, invasion, and migration by modulating the Wnt/β-catenin pathway [14]. Similarly, Zhang et al. [15] reported that silenced Six3 contributes to proliferation and invasion of glioma cancer cells, which is also mediated by activation of the Wnt/β-catenin pathway. Besides, researchers found that expression of Six3 is negatively correlated with breast cancer malignancy and further studies on the mechanism revealed that Six3 recruits LSD1/NuRD(MTA3) complex to Wnt1 promoters, thus resulting in a reduction of Wnt1 expression levels in breast cancer [13]. These findings indicate that Six3 might have a role in regulating tumorigenesis of GC, however, the potential interplay between Six3 and Wnt/β-catenin signaling remains indeterminate in GC.

Long noncoding RNA (lncRNA) is a class of noncoding RNAs with a transcriptional length that exceeds 200 nt. LncRNA has no protein-coding ability but does affect different cellular processes [16]. An increasing number of evidence has shown that abnormal expression of lncRNA in many cancers is closely related to the progression, invasion, and metastasis of cancers. Therefore, lncRNA has the potential to be a biomarker for the diagnosis and prognosis of cancer [17]. Studies have confirmed that lncRNA can regulate the proliferation and migration of tumor cells by participating in the regulation of various signaling pathways, such as the Wnt pathway, the p53 pathway, the NF-κB pathway, etc. [18]. Among them, the interplay between lncRNA and Wnt/β-catenin signaling to regulate the progression of GC has gained more and more attention recently. On the one hand, some lncRNAs can inhibit the progression of GC by directly or indirectly restraining the Wnt pathway. For example, LINC02381 may regulate the activity of the Wnt signaling pathway by competitively binding to miR-21, miR-590, and miR-27a [19]. Similarly, overexpressed LINC01314 acts to downregulate KLK4, thus resulting in the decreased expression of Wnt1, β-catenin, cyclin D1, etc. [20]. On the other hand, other lncRNAs are able to activate Wnt/β-catenin signaling to promote GC progression. For instance, LINC00052, demonstrated a carcinogenic role in GC, can interact with β-catenin and SMYD2 to promote β-catenin methylation and maintain its stability, thereby activating the Wnt/β-catenin signaling pathway [21]. SNHG22 silencing exerts tumor-suppressing potentials in GC development via the Wnt/β-catenin pathway by binding to miR-361-3p and downregulating of HMGA1 [22]. Therefore, these findings highlight the importance of the interaction between lncRNA and Wnt/β-catenin signaling in the tumorigenesis and progression of GC.

Located at chromosome 18p11.31, DLGAP1-AS2 is a lncRNA that was first reported in patients with Wilms’ tumor and may serve as a prognostic marker [23]. And researchers also found that DLGAP1-AS2 is significantly upregulated in several cancers such as glioma, hepatocellular carcinoma (HCC), and cholangiocarcinoma (CCA) [24–26]. The reported mechanisms of DLGAP1-AS2 range from competing endogenous RNA (ceRNA) to epigenetic modification, suggesting versatile roles for DLGAP1-AS2 in the regulation of cancer biology. However, it is still unknown to us regarding the expression pattern and the potential role of DLGAP1-AS2 in GC tumorigenesis.

In this study, we firstly performed a systematic analysis of DLGAP1-AS2 expression with data from the TCGA and GEO databases. We also examined the expression pattern of DLGAP1-AS2 in tumorous tissues of GC from our clinic samples. The results demonstrated up-regulation of DLGAP1-AS2 to be associated with advanced disease and poorer overall survival (OS) of GC. Silencing of DLGAP1-AS2 restricted cell proliferation, migration, and invasion in GC cell lines. Furthermore, DLGAP1-AS2 was found to directly interact with the transcriptional repressor Six3, and this interaction hampered Six3 binding to the promoter regions of the Wnt1 gene, thereby leading to transcriptional activation of Wnt1. Collectively, our findings support an oncogenic role of DLGAP1-AS2 in GC progression and suggest that DLGAP1-AS2/Six3/Wnt1/β-catenin signaling axis may be a potential target for diagnosis and treatment of GC.

## Results

### Upregulation of DLGAP1-AS2 was associated with advanced disease and poorer OS of GC

**Fig. 1:**
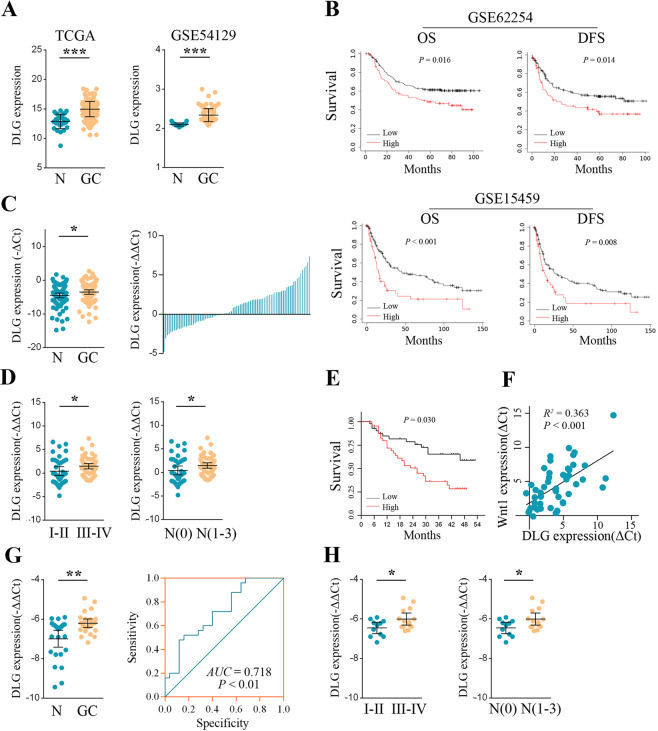
DLGAP1-AS2 was upregulated in GC. **A** Relative expression of DLGAP1-AS2 in GC compared with normal tissue was analyzed by using the TCGA database and GSE54129 dataset. **B** Kaplan–Meier analysis of OS and DFS in GSE662254 and GSE15459 datasets. **C** RT‐qPCR assay for the expression of DLGAP1-AS2 in 85 paired GC tissues and adjacent non-cancerous tissues. **D** The correlation between tissue DLGAP1-AS2 level and TNM stage as well as lymphatic metastasis. **E** Kaplan–Meier analysis of OS according to the median expression level of DLGAP1-AS2 in GC patients. **F** The correlation between DLGAP1-AS2 and Wnt1 expression was determined by Pearson correlation analysis. **G** The expression of DLGAP1-AS2 in plasma from 25 GC patients and paired healthy donors. The accuracy for plasma DLGAP1-AS2 in the diagnosis of GC was analyzed by the ROC curve. **H** The correlation between plasma DLGAP1-AS2 and TNM stage or lymphatic metastasis. DLG: DLGAP1-AS2. ^*^P < 0.05, ^**^P < 0.01, ^***^P < 0.001.

To assess the expression pattern of DLGAP1-AS2 in GC, we compared the data of transcripts per million reads between 375 GC samples and 32 matched non‐cancerous samples from the TCGA database (Fig. 1A). The results showed that DLGAP1-AS2 was significantly upregulated in GC samples. Similarly, we found that DLGAP1-AS2 was highly expressed in GC patients in the GSE54129 dataset (Fig. 1A). We then measured DLGAP1-AS2 expression levels in a cohort of 85 paired GC tissues and adjacent non‐cancerous tissues by reverse transcription-quantitative polymerase chain reaction (RT‐qPCR). As shown in Fig. 1C, GC tissues had significantly higher expression levels of DLGAP1-AS2 than paired non‐cancerous tissues, and 43 cases displayed at least a twofold increase (Fig. 1C). Further analysis of clinical information showed that high expression of DLGAP1-AS2 was significantly correlated with advanced TNM stage and positive lymphatic metastasis (Table 1 and Fig. 1D). Survival analysis showed that patients with high expression of DLGAP1-AS2 exhibited shorter OS and disease-free survival in GSE662254 and GSE15459 datasets, respectively (Fig. 1B). Consistent with these findings, we also observed a poorer OS for patients with high DLGAP1-AS2 expression in our cohort (Fig. 1E). Furthermore, the expression of plasma DLGAP1-AS2 was also higher in GC patients than in healthy controls (Fig. 1G), and its higher expression correlated with advanced disease of GC (Fig. 1H). The receiver operating characteristic (ROC) curve showed that the area under curve value for DLGAP1-AS2 in the diagnosis of GC was 0.718 (95% CI: 0.577–0.860, *P* < 0.01) (Fig. 1G), indicating that plasma DLGAP1-AS2 could be a potential predictor for GC diagnosis. Taken together, these data suggested that DLGAP1-AS2 expression could be a candidate predictor for GC diagnosis and prognosis assessment.

**Table 1:** The correlation between DLGAP1-AS2 expression and the clinicopathological characteristics of GC.

Features	High expression (*n* = 42)	Low expression (*n* = 43)	*P*
*Gender*			
Male	33	31	0.489
Female	9	12	
*Age*			
<60	9	8	0.745
≥60	33	35	
*Drink*			
NO	35	34	0.615
YES	7	9	
*Smoke*			
NO	33	31	0.489
YES	9	12	
*Tumor size*			
< 5 cm	22	28	0.233
≥5 cm	20	15	
*Differentiation*			
Moderate	13	19	0.208
Poor	29	24	
*Lymphatic metastasis*			
Negative	14	24	0.037
Positive	28	19	
*Venous invasion*			
Negative	33	38	0.223
Positive	9	5	
*TNM stages*			
I/II	14	24	0.037
III/IV	28	19	

### Knockdown of DLGAP1-AS2 suppressed proliferation, migration, and invasion of GC cells

**Fig. 2:**
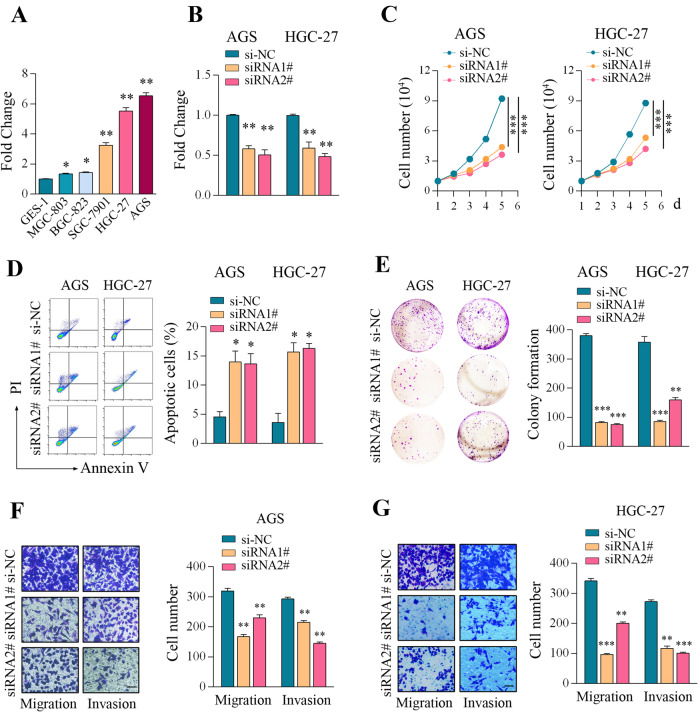
Knockdown of DLGAP1-AS2 suppressed proliferation, migration, and invasion of GC cells. **A** DLGAP1-AS2 expression was measured in a normal gastric epithelial cell line (GES-1) and established GC cell lines (MGC‐803, BGC‐823, SGC‐7901, AGS, and HGC‐27) using RT-qPCR. **B** qPCR verification of the knockdown efficiency of DLGAP1-AS2 by using two independent siRNA oligonucleotides siRNA 1# and siRNA 2# in the treatment of AGS and HGC-27 cells. **C** Cell counting assays upon DLGAP1-AS2 knockdown in GC cells. **D** Apoptotic assays upon DLGAP1-AS2 knockdown in GC cells. **E** Colony formation assays upon DLGAP1-AS2 knockdown in GC cells. **F**, **G** Migration and invasion assays upon DLGAP1-AS2 knockdown. The data shown are representative of three independent experiments. Scale bar: 20 μm. *P < 0.05, **P < 0.01, ***P < 0.001.

We then profiled DLGAP1-AS2 expression in a panel of GC cell lines and found that DLGAP1-AS2 was generally upregulated in the GC cell lines compared with that in GES‐1 (Fig. 2A). We chose the AGS and HGC‐27 cell lines for knockdown experiments because they exhibited the highest expression levels. Two independent siRNAs targeting DLGAP1-AS2, namely siRNA1# and siRNA2#, were designed, and qPCR analyses showed that both siRNA oligonucleotides exhibited reliable silencing efficiency (Fig. 2B). Cell counting assays showed that DLGAP1-AS2 knockdown significantly inhibited cell proliferation of AGS and HGC‐27 cells, respectively (Fig. 2C). The apoptosis assays by flow cytometry showed that DLGAP1-AS2 knockdown induced a significant increase in the apoptotic cells compared with the scrambled negative control in the tested GC cells (Fig. 2D). Colony formation assays and Transwell assays also confirmed that DLGAP1-AS2 silencing markedly reduced the colony number and decreased the migration and invasion ability of GC cells, respectively (Fig. 2E–G). Thus, our findings suggested that DLGAP1-AS2 could drive a malignant phenotype of GC cells by promoting proliferation, invasion, and migration.

### DLGAP1-AS2 modulated the Wnt/β-catenin pathway in GC

**Fig. 3:**
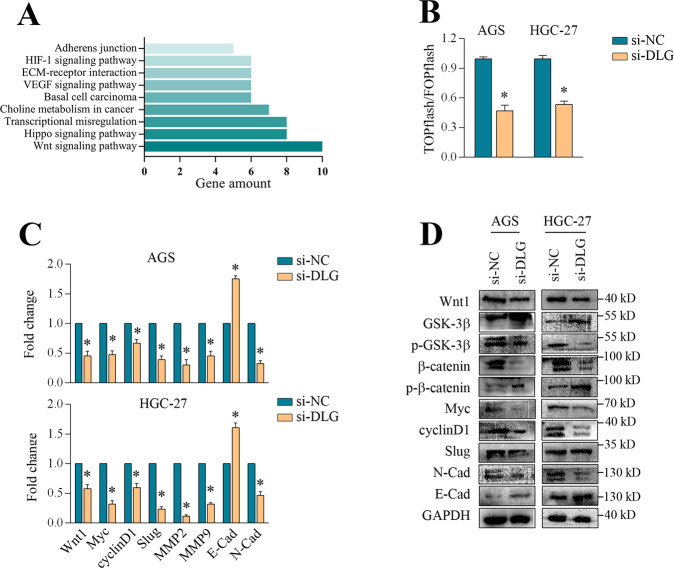
DLGAP1-AS2 modulated the Wnt/β-catenin pathway in GC. **A** KEGG pathway analysis for DLGAP1-AS2 co-expressed genes from TCGA dataset. **B** TOP flash/FOP flash assays in AGS and HGC-27 cells upon DLGAP1-AS2 knockdown. **C** RT-qPCR assays for the expression of Wnt1 and its downstream targets upon DLGAP1-AS2 knockdown. **D** Western blot analysis of the main factors related to Wnt/β-catenin signaling upon DLGAP1-AS2 knockdown. The data shown are representative of three independent experiments. E-Cad E-cadherin, N-Cad N-cadherin. *P < 0.05.

To clarify the potential mechanism of DLGAP1-AS2, we initially analyzed the co-expressed genes of DLGAP1-AS2 from the TCGA dataset and explored their functional enrichment by GO and KEGG analysis using the DAVID website tool. According to the KEGG pathway results, DLGAP1-AS2 was potentially associated with multiple oncogenic pathways including HIF-signaling, VEGF signaling, Hippo signaling, and Wnt signaling, among many others (Fig. 3A). GO analysis revealed that the associated genes of DLGAP1-AS2 were most highly enriched in the GO terms including positive regulation of transcription (biological process) and protein binding (molecular function) (Supplementary Fig. 1A). These findings raised the possibility that DLGAP1-AS2 might function as a transcriptional regulator via RNA–protein interaction, and that the Wnt signaling pathway might be a candidate downstream target of DLGAP1-AS2. To verify this hypothesis, we assessed the expression of a panel of tumor-associated genes that ranged from cell proliferation, apoptosis, epithelial–mesenchymal transition (EMT), and angiogenesis modulation upon DLGAP1-AS2 silencing. The results showed that only Wnt1 and its downstream targets including c-Myc, cyclin D1, Slug, MMP2, MMP9, E-Cadherin, and N-Cadherin were tightly controlled by DLGAP1-AS2 (Fig. 3C and Supplementary Fig. 1B). Consistently, the qPCR analysis in 48 GC tissues also demonstrated a significant correlation between DLGAP1-AS2 and Wnt1 expression (*R*^2^ = 0.363, *P* < 0.001) (Fig. 1F). TOP/FOP luciferase reporter assays are widely used to measure β-catenin-driven transcription [27]. In agreement with suppression in Wnt signaling, our results confirmed that DLGAP1-AS2 silencing led to decreased TCF/LEF activity in GC cells (Fig. 3B). We also examined the expression of other main Wnt members upon DLGAP1-AS2 silencing to rule out the possibility that other Wnt ligands might contribute to this process. The results further indicated that Wnt1 rather than Wnt2B, Wnt3A, Wnt4, Wnt5B, Wnt7A, Wnt9A, and Wnt10B was the unique downstream target of DLGAP1-AS2 (Supplementary Fig. 1C, D). To further understand the change of Wnt/β-catenin signaling upon DLGAP1-AS2 silencing, we measured the key components of Wnt/β-catenin signaling at protein levels. Consistent with our qPCR results, we observed that DLGAP1-AS2 silencing resulted in a marked reduction of Wnt1, which subsequently led to a decrease in phosphorylation (Ser9) of GSK-3β and an increase in GSK-3β stability (Fig. 3D). Thus, β-catenin exhibited a high level of phosphorylation (Ser37) (inactive form) along with high instability (Fig. 3D). It is not surprising that the downstream targets of β-catenin such as Myc, cyclin D1, Slug, and N-cadherin consistently showed marked reduction after DLGAP1-AS2 silencing (Fig. 3D). In summary, these data suggested that DLGAP1-AS2 loss in GC cells attenuated Wnt/β-catenin signaling in GC, and Wnt1 downregulation might critically contribute to this process.

### DLGAP1-AS2 binding to Six3 relieved its occupancy in Wnt1 promoter regions

**Fig. 4:**
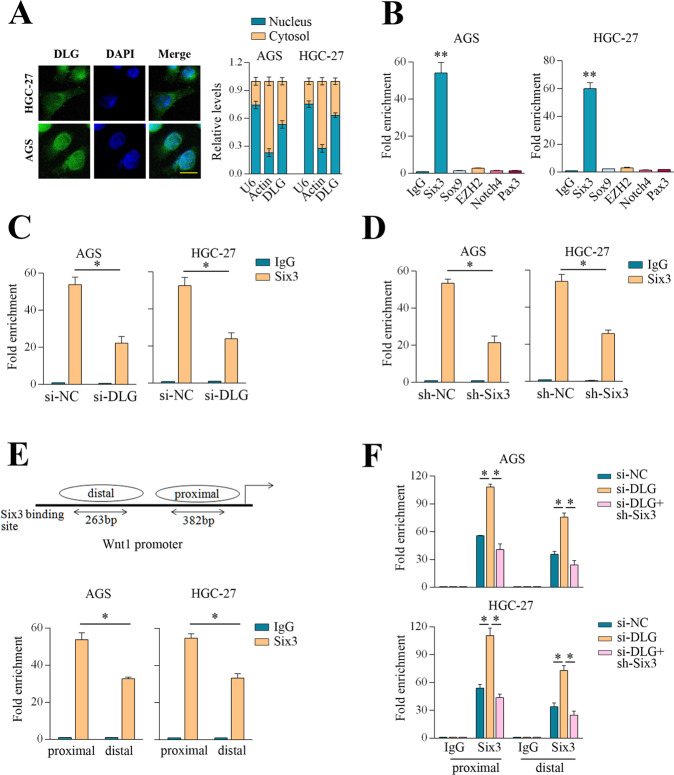
DLGAP1-AS2 binding to Six3 relieved its occupancy in Wnt1 promoter regions. **A** FISH and fractionation assays for the subcellular location of DLGAP1-AS2 in AGS and HGC-27 cells. **B** RIP assays for the potential interaction between DLGAP1-AS2 and several transcriptional regulators that targeted Wnt1. **C**, **D** RIP assays for the binding between DLGAP1-AS2 and Six3 upon DLGAP1-AS2 or Six3 knockdown in GC cells. **E** Schematic representation of Six3 binding sites in Wnt1 promoter (upper). ChIP-qPCR assays showing occupancy of Six3 in both Wnt1 promoter regions (proximal and distal) in GC cells (lower). **F** ChIP-qPCR assays showing differential recruitment of Six3 to both Wnt1 promoter regions upon DLGAP1-AS2 knockdown or combined knockdown of DLGAP1-AS2 and Six3 in GC cells. The data shown are representative of three independent experiments. Scale bar: 10 μm. ^*^P < 0.05. ^**^P < 0.01.

Although we had shown that DLGAP1-AS2 regulated Wnt1 expression, the underlying mechanisms were still unknown. To address this issue, we firstly analyzed the subcellular distribution of DLGAP1-AS2 by the lncATLAS website tool. The results demonstrated that most of DLGAP1-AS2 were predicted to be located in the nucleus in various cell lines, which was in contrast with the location of H19 a cytoplasmic gene, and NEAT1 a nuclear gene (Supplementary Fig. 2A). We also employed fluorescence in situ hybridization (FISH) and subcellular fractionation techniques to verify the distribution of DLGAP1-AS2 in AGS and HGC-27 cells. Consistently, the results showed that DLGAP1-AS2 resided in both the nucleus and cytoplasm of GC cells, with nucleus fraction being more abundant (Fig. 4A). Therefore, it was reasonable to hypothesize that DLGAP1-AS2 might regulate Wnt1 expression at the transcriptional level. Previous studies have shown that several transcription factors and chromatin modifiers such as Six3, pax3, sox9, Notch4, and EZH2 may regulate Wnt1 expression [13, 28–31]. We then tested their potential interactions with DLGAP1-AS2 by RIP assays in GC cells. The results showed that endogenous DLGAP1-AS2 was enriched in the anti-Six3 RIP fraction relative to the input compared to the anti-pax3, sox9, Notch4, or EZH2 fraction (Fig. 4B). This finding suggested that DLGAP1-AS2 could specifically bind to Six3 rather than the other transcription factors or chromatin modifiers. Furthermore, the binding efficiency between DLGAP1-AS2 and Six3 was impaired when either DLGAP1-AS2 or Six3 was silenced (Fig. 4C, D). Previous studies have well demonstrated that Six3 can specifically bind to the Wnt1 promoter regions, namely −2074 to −2456 bp promoter region (proximal) and −3324 to −3857 bp enhancer element (distal) [32]. In the chromatin immunoprecipitation (ChIP)-qPCR assays, we used primers encompassing 382 bp of the Wnt1 5′-promoter region and also 263 bp of the Wnt1 enhancer element (Fig. 4E). Consistent with previous findings in breast cancer cells, we observed an enrichment of Six3 to the promoter regions of Wnt1 compared to the corresponding IgG control in AGS and HGC-27 cells (Fig. 4E). The binding of Six3 to Wnt1 promoter regions may restrain the activation of Wnt1 transcription [32]. In agreement with this, we observed increased expression of Wnt1 expression and enhanced TCF/LEF activity in Six3 knockdown GC cells (Supplementary Fig. 2E). Moreover, silencing of DLGAP1-AS2 could enhance the binding of Six3 to the Wnt1 promoter regions (Fig. 4F). It might also be possible that DLGAP1-AS2 regulated Wnt1 expression via altering Six3 expression or degradation. To rule out this possibility, Six3 expression or protein stability was examined in DLGAP1-AS2 knockdown cells. The results showed that silencing of DLGAP1-AS2 had no impact on Six3 mRNA expression or protein levels (Supplementary Fig. 2B, C). DLGAP1-AS2 deficiency either did not alter the stability and half-life of Six3 in CHX treated GC cells (Supplementary Fig. 2D). Therefore, these findings highlighted a competitive interaction mechanism between DLGAP1-AS2, Six3, and Wnt1 promoter regions, and suggested that the binding of DLGAP1-AS2 to Six3 might relieve its occupancy in Wnt1 promoter regions.

### Six3 contributed to DLGAP1-AS2-mediated Wnt/β-catenin signaling

**Fig. 5:**
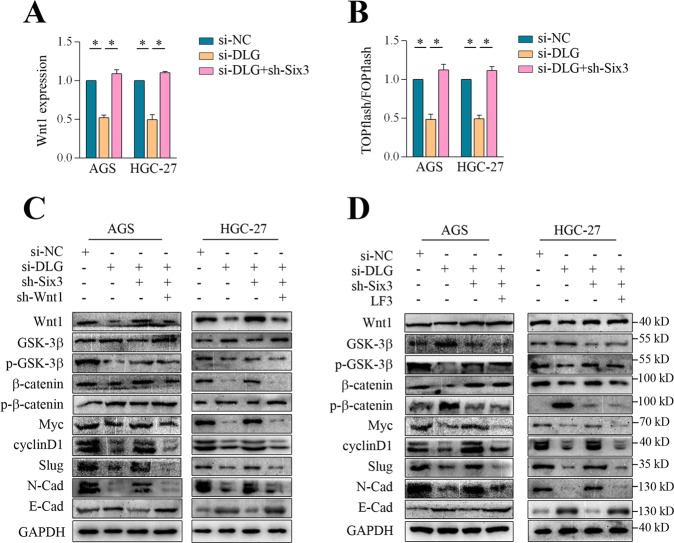
Six3 and Wnt1 contributed to DLGAP1-AS2 mediated Wnt/β-catenin signaling. **A** RT-qPCR analysis of Wnt1 expression in GC cells transfected with si-NC, si-DLGAP1-AS2, and si-DLGAP1-AS2 + sh-Six3. B TOP/FOP assays showing differential TCF/LEF activity between si-NC, si-DLGAP1-AS2, and si-DLGAP1-AS2 + sh-Six3 groups. C Western blot analysis of the main factors related to Wnt/β-signaling upon transfection of si-NC, si-DLGAP1-AS2, si-DLGAP1-AS2 + sh-Six3, and si-DLGAP1-AS2 + sh-Six3+sh-Wnt1. D Western blot analysis of the main factors related to Wnt/β-signaling upon transfection/treatment with si-NC, si-DLGAP1-AS2, si-DLGAP1-AS2 + sh-Six3, and si-DLGAP1-AS2 + sh-Six3 + LF3. The data shown are representative of three independent experiments. ^*^P < 0.05.

**Fig. 6:**
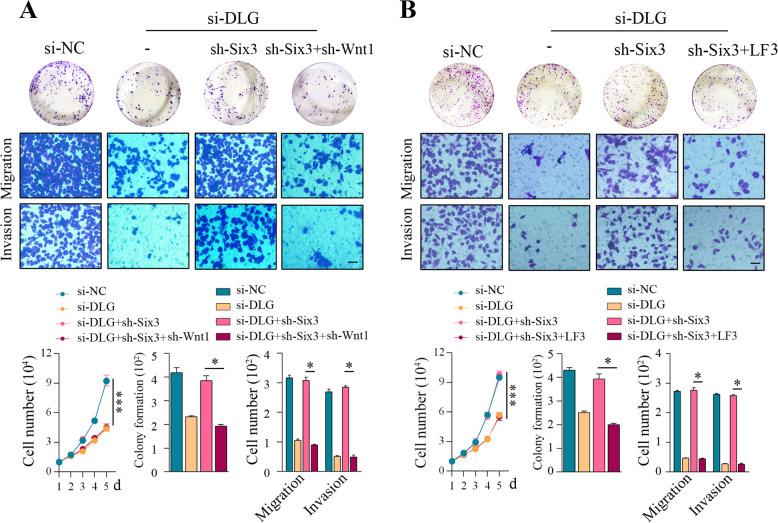
Inhibition of Wnt1 reversed the malignant phenotypes of GC driven by Six3 depletion. **A** Colony formation assays, migration assays, invasion assays, and cell proliferation assays in AGS cells upon transfection of si-NC, si-DLGAP1-AS2, si-DLGAP1-AS2 + sh-Six3, or si-DLGAP1-AS2 + sh-Six3+sh-Wnt1. **B** Colony formation assays, migration assays, invasion assays, and cell proliferation assays in AGS cells upon transfection/treatment with si-NC, si-DLGAP1-AS2, si-DLGAP1-AS2 + sh-Six3, or si-DLGAP1-AS2 + sh-Six3 + LF3. The data shown are representative of three independent experiments. Scale bar: 20 μm. ^*^P < 0.05, ^***^P < 0.001.

To further ascertain the role of Six3 in DLGAP1-AS2-mediated Wnt1 transcriptional activation, we measured Wnt1 expression and TCF/LEF activity in GC cells corresponding to DLGAP1-AS2 and Six3 knockdown. The results showed that the knockdown of Six3 totally reversed the DLGAP1-AS2 deficiency-associated reduction in Wnt1 expression and TCF/LEF activity (Fig. 5A, B). Consistently, Western blot assays showed that DLGAP1-AS2 deficiency-induced suppression in Wnt1, β-catenin, Myc, cyclinD1, Slug, and N-cadherin were all totally reversed by Six3 knockdown (Fig. 5C, D). Therefore, it was not surprising that knockdown of Six3 was effective to reverse the attenuated GC malignancy by DLGAP1-AS2 depletion, leading to enhanced proliferation, colony formation, migration, and invasion (Fig. 6A, B, Supplementary Fig. 3A, B). These findings supported a pivotal role of Six3 in DLGAP1-AS2 mediated activation of Wnt/β-catenin signaling and malignant transformation of GC.

### DLGAP1-AS2 mediated Wnt/β-catenin signaling dependent on Wnt1

To verify the role of Wnt1 in DLGAP1-AS2 mediated Wnt/β-catenin signaling, we employed two independent procedures to inhibit Wnt1 signaling in GC cells, which were Wnt1 knockdown and β-Catenin/TCF4 inhibition (LF3), respectively. Although Six3 knockdown reversed the DLGAP1-AS2 mediated attenuation in Wnt/β-catenin signaling, either genetic or pharmacological inhibition of Wnt1 signaling rescued the above effect, thereby restraining the activation of Wnt/β-catenin downstream targets and conferring a retarded phenotype of GC cells (Fig. 5C, D, Fig. 6A, B, Supplementary Fig. 3A, B). These findings suggested that DLGAP1-AS2 mediated Wnt/β-catenin signaling is dependent on Wnt1.

### DLGAP1-AS2 regulated GC progression via Six3/Wnt1/β-catenin signaling pathway in vivo

**Fig. 7:**
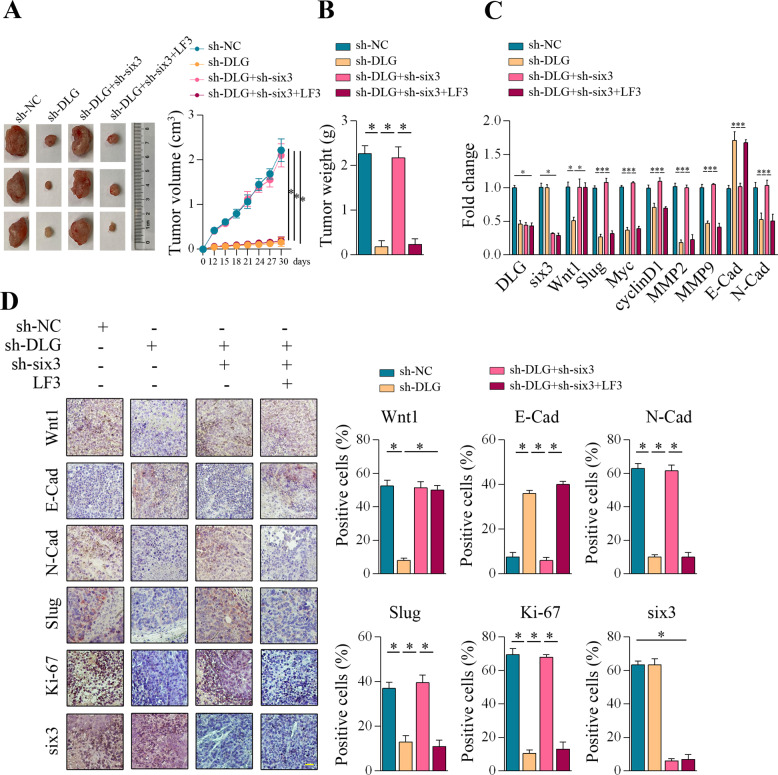
DLGAP1-AS2 regulated GC progression via Six3/Wnt1/β-catenin signaling pathway in vivo. **A** Tumor samples and growth curves from sh-NC, sh-DLGAP1-AS2, sh-DLGAP1-AS2 + sh-Six3, and sh-DLGAP1-AS2 + sh-Six3 + LF3 groups were shown (*n* = 6 for each group). **B** Tumor weight from the respective groups was represented. **C** RT-qPCR analysis of DLGAP1-AS2, Six3, Wnt1, Slug, Myc, cyclinD1, MMP2, MMP9, E-cadherin, and N-cadherin in xenograft tumors for each group. **D** IHC assays of Wnt1, E-cadherin, N-cadherin, Slug, Ki-67, and Six3 in paraffin tumor sections for each group. Scale bar: 50 μm. ^*^P < 0.05.

To determine whether DLGAP1-AS2 affected tumor formation in vivo, nude mice were injected with DLGAP1-AS2 knockdown AGS cells. To further study the contribution of Six3 in this process, Six3 knockdown cells on the background of DLGAP1-AS2 depletion were also generated and injected into nude mice. For Wnt/β-Catenin signaling inhibition, LF3 was administered 10 days post tumor implantation. Consistent with in vitro results, tumor growth in the DLGAP1-AS2 knockdown group was obviously slower than that in the scrambled control group (Fig. 7A). Knockdown of Six3 completely abolished the DLGAP1-AS2 deficiency mediated effect, which could be further reversed by LF3 (Fig. 7A). Tumor weight comparison also yielded similar results (Fig. 7B). RT-qPCR analysis verified the knockdown efficiency of DLGAP1-AS2 and Six3 in the xenograft tumors (Fig. 7C). Importantly, DLGAP1-AS2 knockdown led to suppression of Wnt1, Slug, Myc, cyclinD1, MMP2, MMP9, and N-cadherin in qPCR and/or IHC assays, and reduction of proliferation marker Ki-67 (Fig. 7C, D). Again, the knockdown of Six3 completely abolished the DLGAP1-AS2 deficiency mediated effects, which could be further reversed by LF3 (Fig. 7C, D). In summary, these data indicated an oncogenic role of DLGAP1-AS2 in tumorigenesis and progression of GC and suggested that Six3/Wnt1/β-catenin signaling acted downstream of DLGAP1-AS2 to precisely control this process.

## Discussion

Among the recent GC research works, the vital role of lncRNAs in tumor biology has gained more and more attention with the help of improved sequencing techniques, microarray techniques, bio-information analyses, and insightful mechanism researches. Acting either as oncogene or tumor suppressor, many lncRNAs have been shown to be implicated in the regulation of GC carcinogenesis and progression. However, the specific mechanism between lncRNAs and the malignancy of GC was still incompletely understood. In this study, we aimed to explore the role of DLGAP1-AS2 in GC development, which has shown to be significantly increased in GC tumorous tissues, plasmas, and cell lines. Our findings revealed the potential link between high expression of DLGAP1-AS2 and advanced disease as well as poorer survival of GC, suggesting that DLGAP1-AS2 may be utilized as a diagnostic and/or prognostic indicator for GC.

By conducting combined functional assays such as cell proliferation assay, clonogenic assay, apoptotic assay, Transwell assay, and xenograft model, we were able to demonstrate that DLGAP1-AS2 drove the malignancy and progression of GC. Generally considered as an oncogenic factor, DLGAP1-AS2 has recently been reported to modulate the tumorigenesis and progression of several tumors, such as HCC [24], CCA [25], and Glioma [26]. The reported mechanisms of DLGAP1-AS2 range from ceRNA to epigenetic modification, suggesting versatile roles for DLGAP1-AS2 in the regulation of cancer biology. However, it is still unknown to us regarding the potential role of DLGAP1-AS2 in GC tumorigenesis. To address this issue, we initially performed combined procedures including bioinformatics analysis and multiple gene screening, and ultimately identified Wnt/β-catenin signaling as the downstream target of DLGAP1-AS2. Depletion of DLGAP1-AS2 led to consistent suppression of Wnt1 and its downstream target including β-catenin, c-Myc, cyclin D1, Slug, and N-cadherin. TOP/FOP assay also verified the suppression of Wnt/β-catenin signaling upon DLGAP1-AS2 knockdown. It is well accepted that the Wnt/β-catenin signaling pathway played a critical role in the initiation of and/or maintenance and development of many cancers [33], and it has been found closely associated with the progression of GC [34]. Activation of Wnt/β-catenin signaling leads to accumulation of β-catenin and ultimately translocation to the nucleus where it combines with the TCF/LEF complex and activates transcription target genes. The known downstream targets of β-catenin, including MMP-9, c-Myc, cyclin D1, etc., are mainly responsible for β-catenin-driven malignancy in cell proliferation, cell cycle progression, and metastasis in cancer biology.

Emerging evidence has supported the crucial role of lncRNAs in the regulation of the Wnt signaling cascade at both transcriptional and translation levels. The interplay between lncRNA and Wnt signaling cascade is brimming with opportunities to delineate the role of these micromanagers in tumorigenesis of GC. For instance, lncRNA GATA6-AS1 reduces FZD4 expression by recruiting EZH2 and H3K27me3 to the FZD4 promoter region, thereby inactivating the Wnt/β-catenin signaling pathway and ultimately inhibiting GC progression [35]. LINC01133 has been shown to regulate the Wnt/β-catenin pathway by acting as a ceRNA for miR-106a-3p and inhibit GC progression and metastasis [36]. We have also reported that LINC01225 depletion inhibits Wnt/β-catenin signaling and EMT process of GC, an effect abolished by ectopic expression of Wnt1 or suppression of GSK-3β. To gain insight into the potential mechanism by which DLGAP1-AS2 regulated Wnt1, we firstly examined the intracellular localization of DLGAP1-AS2 in GC cells. The results showed that DLGAP1-AS2 distributed in both the nucleus and cytoplasm, with nucleus fractionation more abundant. This is also consistent with the location prediction results by lncATLAS, and points toward the possibility of DLGAP1-AS2 to act as a transcriptional regulator. Previous studies have shown that lncRNA could form an RNA–protein complex to modulate gene transcription in tumor biology, of which the binding of lncRNA with several epigenetic modifiers such as EZH2 is most frequently reported [37–39]. In the present study, we identified Six3 as a new binding cofactor of DLGAP1-AS2 by conducting a RIP assay among the known transcription regulators that could potentially control Wnt1 expression.

Six3 is a member of the Six family of homeoproteins that share a remarkable similarity to a common binding sequence (TCAGGTTC) in mammals [13]. As a DNA-binding transcription factor Six3 is downregulated in lung cancer and correlated with tumor size, OS, and recurrence of patients with lung adenocarcinoma [40]. Of note, the Wnt/β-catenin signaling pathway is involved in Six3 function in glioma and breast cancer development [15, 41]. This was supported by our observation that Six3 bound to the Wnt1 promoter in GC cells and repressed Wnt1 expression. Our finding that DLGAP1-AS2 and Six3 function as an integral RNA–protein complex marks the first time that DLGAP1-AS2 has been identified as a component of a complex exerting control over the transcriptional activity. Although Six3 has previously been shown to target a group of Wnt ligands including Wnt1, Wnt3, and Wnt5A via interaction with LSD1/NuRD(MTA3) [13], our findings showed no change in the expression of other Wnt members except for Wnt1 upon DLGAP1-AS2 silencing. It might be possible that the Six3 interacting with different cofactors could change the specificity and function of Six3, and ultimately result in different downstream targets activation. In addition, we observed that DLGAP1-AS2 mediated Wnt/β-catenin signaling dependent on Wnt1. Although Six3 knockdown reversed the attenuation in Wnt/β-catenin signaling by DLGAP1-AS2 depletion, either genetic or pharmacological inhibition of Wnt1 signaling rescued the above effect, thereby restraining the activation of Wnt/β-catenin signaling and conferring an ameliorated phenotype of GC cells.

In conclusion, we demonstrate that high expression of DLGAP1-AS2 in GC patients correlates with advanced diseases and predicts a poorer prognosis of GC. DLGAP1-AS2 silencing results in decreased expression of Wnt1 and attenuated Wnt/β-catenin signaling, which contributes to the attenuation of GC malignancy. We also find that the interaction between DLGAP1-AS2 and Six3 is crucial for Six3 dissociation from Wnt1 promoter regions and consequently Wnt1 transcriptional activation. Therefore, our findings of DLGAP1-AS2 binding to Six3 to relieve its repression on Wnt1 transcription thus expanded our understanding of the role of DLGAP1-AS2 and Six3 in the regulation of tumor biology, and support the pursuit of DLGAP1-AS2/Six3/Wnt1/β-catenin as a potential prognostic indicator and therapeutic targets for GC.

## Materials and methods

### Data extraction and analysis

The Cancer Genome Atlas (TCGA) and Gene Expression Omnibus (GEO) database were selected to learn the expression pattern of DLGAP1-AS2 in GC initially. Briefly, raw RNA sequencing data containing 375 gastric adenoma and adenocarcinoma samples and 32 matched non‐cancerous samples were obtained from the TCGA stomach adenocarcinoma (STAD) dataset. The data were then normalized and analyzed with the edgeR package. The co-expressed genes (logFC > 2) of DLGAP1-AS2 were then used for the Gene Ontology (GO) and Kyoto Encyclopedia of Genes (KEGG) enrichment analysis by the DAVID website (http://david.ncifcrf.gov/). Microarray dataset (GSE54129) containing transcriptome data of 111 GC samples and 21 non‐cancerous samples was downloaded from the GEO database and normalized using Robust Multichip Average (RMA) method. Differential analysis was conducted using the limma package. The dataset from Kaplan-Meier Plotter (http://kmplot.com) was used to analyze the clinical outcomes of GC in relation to DLGAP1-AS2. The location of DLGAP1-AS2 was predicted using the database lncATLAS (http://lncatlas.crg.edu).

### Patients and specimens

A total of 85 pairs of GC tissues and their corresponding non‐cancerous tissues (>5 cm away from the edge of GC) were obtained from patients who underwent surgical resection in the Affiliated People’s Hospital of Jiangsu University between 2015 and 2019. No patients had undergone chemoradiotherapy before surgery, and their tissues were pathologically confirmed. The collected tissues were immediately placed in liquid nitrogen for temporary preservation and stored at −80 °C for long-term storage. Plasma samples were collected from the same cohort of patients as well as 25 healthy donors during the same period. Patient clinic information was collected from the medical record, pathological report, and follow-up data. The tumor stage was evaluated according to the eighth TNM staging of the International Union against Cancer (UICC)/American Joint Committee on Cancer (AJCC) system. This study was approved by the Institutional Ethical Committee of Jiangsu University and signed informed consents were obtained from all patients.

### Cell culture and transfection

Five human GC cell lines SGC‐7901, MGC‐803, HGC‐27, AGS, and BGC‐823 were obtained from the Institute of Biochemistry and Cell Biology of the Chinese Academy of Sciences (Shanghai, China). The human normal gastric epithelial cell line (GES‐1) was purchased from Procell Life Science & Technology. MGC‐803 was cultured with Dulbecco’s modified Eagle’s medium (DMEM; Thermo Fisher Scientific), while SGC‐7901, HGC‐27, AGS, BGC‐823, and GES‐1 cells were cultured with Roswell Park Memorial Institute (RPMI) 1640 medium (Thermo Fisher Scientific). All media were supplemented with 10% fetal bovine serum (FBS; Gibco), 100 U/mL penicillin, and 100 μg/mL streptomycin (Gibco) in humidified air at 37 °C with 5% CO_2_.

### RNA isolation and RT‐qPCR

Total RNA was extracted using TRIzol Reagent (Sangon Biotech) and reversely transcribed into cDNA with Hieff First Strand cDNA Synthesis SuperMix for RT‐qPCR kit (Yeasen Biotech). qPCR analysis was carried out using the Hieff qPCR SYBR Green Master Mix kit (Yeasen Biotech) on ABI 7500 real‐time PCR system (AppliedBiosystems). The primer sequences were listed in Supplementary Table 1. β‐actin or U6 was used as an endogenous control depending on the gene/sample detected. Relative expression levels were calculated by using the 2^−ΔΔCt^ method.

### Oligonucleotides, constructs, and treatments

Small interfering RNA (siRNA) oligonucleotides targeting DLGAP1-AS2 were designed and synthesized by GenePharm Company. Six3 and Wnt1 knockdown plasmid were constructed by GenePharm. The targeted sequences were listed in Supplementary Table 1. For transfection, the cells were grown in a 12‐well plate until confluence at 60–80% and were transfected with the indicated molecules with Lipofectamine 2000 (Thermo Fisher Scientific). For pharmacological inhibition of the β-Catenin/TCF4 interaction, the cells were treated with LF3 (30 μM) for 24 h as previously described [42]. To examine whether DLGAP1-AS2 regulated the Six3 protein stability, Cycloheximide (CHX) (30 μg/mL) was used to treat DLGAP1-AS2 knockdown cells for different time periods and the abundance of Six3 was detected.

### Cell proliferation, colony formation, and apoptosis

For proliferation assay, GC cells were seeded into 24‐well plates at a density of 1 × 10^4^ cells/well. The cells were collected and counted every day for 6 days. The results were plotted as cell growth curves. For colony formation assay, the cells were plated in 6‐well plates (1000 cells/well) and were cultured for 10 days. The colonies were then washed, fixed, and stained with 0.5% crystal violet. Visible colonies were counted and photographed. For cell apoptosis assay, the indicated cells were stained with Annexin V‐FITC/PI cell apoptosis detection kit (BD Pharmingen) and analyzed with a flow cytometer (BD FACSCalibur). Experiments were carried out in triplicate independently.

### Migration and invasion assay

Migration and invasion assay was performed in Transwell chambers without or with Matrigel‐coated membranes, respectively. Briefly, GC cells were seeded with serum‐free medium into the upper chambers at 5 × 10^4^ cells/well, and the bottom chambers contained a medium with 10% FBS. After culturing for 24 h, cells on the upper surface were removed with a cotton swab and cells on the lower surface were stained and counted under a microscope.

### Western blot

Equal amounts of protein samples were separated by 8–15% sodium dodecyl sulfate-polyacrylamide gel electrophoresis and transferred to nitrocellulose membranes. Bands were probed immunologically using anti-Six3 (Santa Cruz), GSK‐3β (EnoGene), Phospho‐GSK‐3β (Ser9) (EnoGene), β‐catenin (EnoGene), Phospho-β‐catenin (Ser37) (EnoGene), Slug (EnoGene), E‐cadherin (EnoGene), N‐cadherin (EnoGene), c-Myc (EnoGene), and cyclin D1 (EnoGene). GAPDH (Cell signaling) was probed as an internal reference. Signals were detected using an enhanced chemiluminescence (ECL) system according to the manufacturer’s instructions.

### TOP/FOP-flash luciferase assay

A pair of luciferase reporter constructs, TOP-FLASH reporter gene, and FOP-FLASH control construct were used to evaluate TCF/LEF transcriptional activity. Briefly, cells were transiently transfected in triplicate with one of the luciferase reporters and phRL-TK (internal control). The transfected cells were then harvested, and luciferase activity was measured using the Dual-Luciferase Reporter Assay System (Promega). Data are presented as the ratio of TOP-FLASH to FOP-FLASH (TOP/FOP ratio).

### RNA immunoprecipitation (RIP)

RIP was used to investigate whether DLGAP1-AS2 could interact or bind with the potential cofactors in GC cells. We used the EZMagna RIP kit (Millipore, Billerica, MA, USA) following the manufacturer’s protocol. Briefly, AGS and HGC-27 cells were lysed in complete RIP lysis buffer, and the extract was incubated with magnetic beads conjugated with antibodies that recognized Six3 (Santa Cruz), pax3 (Santa Cruz), sox9 (Santa Cruz), Notch4 (Santa Cruz), EZH2 (Cell signaling), or control IgG (Santa Cruz) for 6 h at 4 °C. Then, the beads were washed and incubated with Proteinase K to remove proteins. Finally, purified RNA was subjected to RT-qPCR analysis to demonstrate the presence of DLGAP1-AS2 using specific primers.

### Subcellular fractionation

The separation of nuclear and cytosolic fractions was performed using the PARIS Kit (Life Technologies) according to the manufacturer’s instructions. RT-qPCR was conducted to determine the relative enrichment of DLGAP1-AS2 in cytoplasmic or nuclear components. β-actin and U6 were served as cytoplasmic and nuclear reference, respectively.

### Fluorescence in situ hybridization (FISH)

The FAM-labeled DLGAP1-AS2 probes were designed and synthesized by GenePharm, and FISH was conducted using the FISH kit (GenePharm) according to the manufacturer’s protocol. Cell nuclei were then counterstained with 4′,6-diamidino-2-phenylindole (DAPI) and observed under a laser scanning confocal microscope from Leica.

### Chromatin immunoprecipitation (ChIP)

AGS and HGC-27 cells were treated with formaldehyde and incubated for 10 min to generate DNA-protein cross-links. Cell lysates were then sonicated to generate chromatin fragments of 200–300 bp and immunoprecipitated with Six3 specific antibody (Santa Cruz) or IgG (Santa Cruz) as control. Precipitated chromatin DNA was recovered and analyzed by qPCR to demonstrate the presence of Wnt1 promoter fragments. The primers designed for the detection of Wnt1 promoter regions were listed in Supplementary Table 1.

### Immunohistochemistry (IHC)

Briefly, tissue sections were sequentially treated following the procedures of deparaffinage, rehydration, and antigen retrieval. Endogenous peroxidase was blocked by incubation in 3% H_2_O_2_ for 10 min at room temperature. The sections were then incubated with anti‐Ki‐67 (Sangon), E‐cadherin (EnoGene), or N‐cadherin (EnoGene) antibodies overnight at 4 °C. Finally, HRP‐conjugated secondary antibody and diaminobenzidine (DAB) solution (Solarbio) were used to detect the signals. Slides were photographed under a microscope. The results were represented by the ratio of positively labeled cells.

### Tumor xenograft experiment

Four‐week‐old female athymic BALB/c mice were purchased from the Experimental Animal Center of the Chinese Academy of Sciences (Shanghai, China) and maintained under specific pathogen‐free conditions. The validated DLGAP1-AS2 interference sequence was firstly subcloned into pLKO.1 vector to generate sh‐DLGAP1-AS2 plasmid. AGS cells transfected with sh‐DLGAP1-AS2 or sh‐control were selected by puromycin (Sigma‐Aldrich). To block the DLGAP1-AS2 deficiency-mediated effects, DLGAP1-AS2-silencing cells were transfected with Six3 shRNA and selected with hygromycin B (Sigma‐Aldrich) in some groups. Approximately, 2 × 10^6^ AGS cells were resuspended with 0.2 mL phosphate buffer saline and injected into the dorsal right flank of each nude mouse (six mice per group). The grouping was performed randomizedly. Tumor volumes were calculated as 0.5 × length × width^2^ every 3 days. For Wnt/β-Catenin signaling inhibition, LF3 was administered i.v. at 50 mg/kg body weight for three rounds over 5 consecutive days, with 2-day breaks, since 10 days post tumor implantation. After 30 days, the mice were killed, and tumors were excised, weighed, and immediately fixed with formaldehyde or frozen at −80 °C for future use. The procedures for animal studies were approved by the Animal Use and Care Committee of Jiangsu University.

### Statistical analysis

All statistical analyses were conducted with SPSS 24.0 software (IBM, SPSS). Student’s *t* tests or one‐way analysis of variance (ANOVA) was performed to determine the significance of differences between different groups. The associations between DLGAP1-AS2 and the clinicopathological features were analyzed by the Pearson chi-squared test. Survival curves according to DLGAP1-AS2 expression were generated using the Kaplan–Meier method, and their difference was evaluated by log‐rank test. ROC curve was established to evaluate the diagnostic value of DLGAP1-AS2 for GC. A two‐sided *P* value < 0.05 was considered statistically significant.

## Supplementary information

Supplementary Table 1

Supplementary Figure 1

Supplementary Figure 2

Supplementary Figure 3

Supplementary Figure legend

## Data Availability

All data obtained and/or analyzed during the current study are available from the corresponding authors on reasonable request.
