# Supplementary material for: Long noncoding RNA DLGAP1-AS2 facilitates Wnt1 transcription through physically interacting with Six3 and drives the malignancy of gastric cancer
Source: Cell Death Discov. 2021 Sep 20;7:255. doi: 10.1038/s41420-021-00649-z (PMC8452735; doi:10.1038/s41420-021-00649-z)
Supplement: Supplementary file 1 — Supplementary Table 1 [file 41420_2021_649_MOESM1_ESM.docx]

**Supplementary Table 1. Primer or silencing sequences for the indicated genes**

| Gene name | Sequence |
| --- | --- |
| DLGAP1-AS2-F | CTTCAACTCGCCACAGGCTA |
| DLGAP1-AS2-R | GACAGCATCGGGTCAAAGGA |
| Wnt1-F | ATCTTCGCTATCACCTCCGC |
| Wnt1-R | GGCCGAAGTCAATGTTGTCG |
| c-Myc-F | GGACTTGTTGCGGAAACGAC |
| c-Myc-R | CTCAGCCAAGGTTGTGAGGT |
| TWIST1-F | GGAGTCCGCAGTCTTACGAG |
| TWIST1-R | TCTGGAGGACCTGGTAGAGG |
| Slug-F | TGTGACAAGGAATATGTGAGCC |
| Slug-R | TGAGCCCTCAGATTTGACCTG |
| cyclin D1-F | AGGTCTGCGAGGAACAGAAG |
| cyclin D1-R | CCACGAACATGCAAGTGGC |
| E-cad-F | GGTCTGTCATGGAAGGTGCTC |
| E-cad-R | CAGGATCTTGGCTGAGGATGG |
| N-Cad-F | TCAACTGCAACCGTGTCTGT |
| N-Cad-R | ATCGATCTGGGTCCTGAGCA |
| MMP2-F | CATGGCGATGGATACCCCTT |
| MMP2-R | CCATCGGCGTTCCCATACTT |
| MMP9-F | CAAGTGGCACCACCACAACATCA |
| MMP9-R | CCCGCGGCAAGTCTTCCGA |
| CDK2-F | GTACCTCCCCTGGATGAAGAT |
| CDK2-R | CGAAATCCGCTTGTTAGGGTC |
| CDK4-F | CTGGTGTTTGAGCATGTAGACC |
| CDK4-R | GATCCTTGATCGTTTCGGCTG |
| P53-F | GCTCTGACTGTACCACCATCC |
| P53-R | CTCTCGGAACATCTCGAAGCG |
| PTEN-F | GCACTGTTGTTTCACAAGATGATG |
| PTEN-R | GCAGACCACAAACTGAGGATTG |
| Bcl-2-F | CTGGGATGCCTTTGTGGAAC |
| Bcl-2-R | CAGGCATGTTGACTTCACTTGT |
| Bax-F | GTCGCCCTTTTCTACTTTGCC |
| Bax-R | AGTCGCTTCAGTGACTCGG |
| Bcl-xL-F | CCCAGAAAGGATACAGCTGG |
| Bcl-xL-R | GCGATCCGACTCACCAATAC |
| BNIP3-F | TTTAAACACCCGAAGCGCAC |
| BNIP3-R | CTGGTGGAGGTTGTCAGACG |
| VEGF-F | GAGATGAGCTTCCTACAGCACA |
| VEGF-R | TCACCGCCTCGGCTTGTC |
| EGFR-F | CCTGGTCTGGAAGTACGCAG |
| EGFR-R | CGATGGACGGGATCTTAGGC |
| Wnt2B-F | CGGGACCACACCGTCTTTG |
| Wnt2B-R | GCGAGTAATAGCGTGGACTAC |
| Wnt3A-F | CTTTGTCCACGCCATTGCC |
| Wnt3A-R | CCAAACTCGATGTCCTCGCT |
| Wnt4-F | CTCCACACTCGACTCCTTGC |
| Wnt4-R | CCGAAGAGATGGCGTACACG |
| Wnt5B-F | CGCTTCGCCAAGGAGTTTG |
| Wnt5B-R | TGCCATCTTATACACAGCCCT |
| Wnt7A-F | GACGCCATCATCGTCATAGG |
| Wnt7A-R | CCACTTTGAGCTCCTTCCCG |
| Wnt9A-F | CCACCGTGAGAAGAACTGC |
| Wnt9A-R | GCCTGCACTCCACATAGCA |
| Wnt10B-F | GTGAGCGAGACCCCACTATG |
| Wnt10B-R | CACTCTGTAACCTTGCACTCATC |
| β-actin-F | CATTCCAAATATGAGATGCGTTGT |
| β-actin-R | TGTGGACTTGGGAGAGGACT |
| U6-F | CTCGCTTCGGCAGCACA |
| U6-R | AACGCTTCACGAATTTGCGT |
| Wnt1-ChIP-proximal-F | TTCCATCAGGCTTCATTTGG |
| Wnt1-ChIP-proximal-R | ACTTGTCCAAGGTCCACGTC |
| Wnt1-ChIP-distal-F | TCATCCACGGTCAAAACTGA |
| Wnt1-ChIP-distal-R | CTAGCCCTCCAGCTTCATTG |
| si-DLGAP1-AS2 1#-F | CCACCACUCCCUAUGGCUUTT |
| si-DLGAP1-AS2 1#-R | AAGCCAUAGGGAGUGGUGGTT |
| si-DLGAP1-AS2 2#-F | CCAAAUUUGAUGCCAACUUTT |
| si-DLGAP1-AS2 2#-R | AAGUUGGCAUCAAAUUUGGTT |
| si-NC-F | UUCUCCGAACGUGUCACGUTT |
| si-NC-R | ACGUGACACGUUCGGAGAATT |
| sh-Six3 | CCGGCCCGGAAGAGTTGTCCATGTTCTCGAGAACATGGACAACTCTTCCGGGTTTTT |
| sh-Wnt1 | CCGGAATCCTGCACGTGTGACTACCTCGAGGTAGTCACACGTGCAGGATTTTTTT |
