# Supplementary figures and images for: Long noncoding RNA DLGAP1-AS2 facilitates Wnt1 transcription through physically interacting with Six3 and drives the malignancy of gastric cancer
Source: Cell Death Discov. 2021 Sep 20;7:255. doi: 10.1038/s41420-021-00649-z (PMC8452735; doi:10.1038/s41420-021-00649-z)

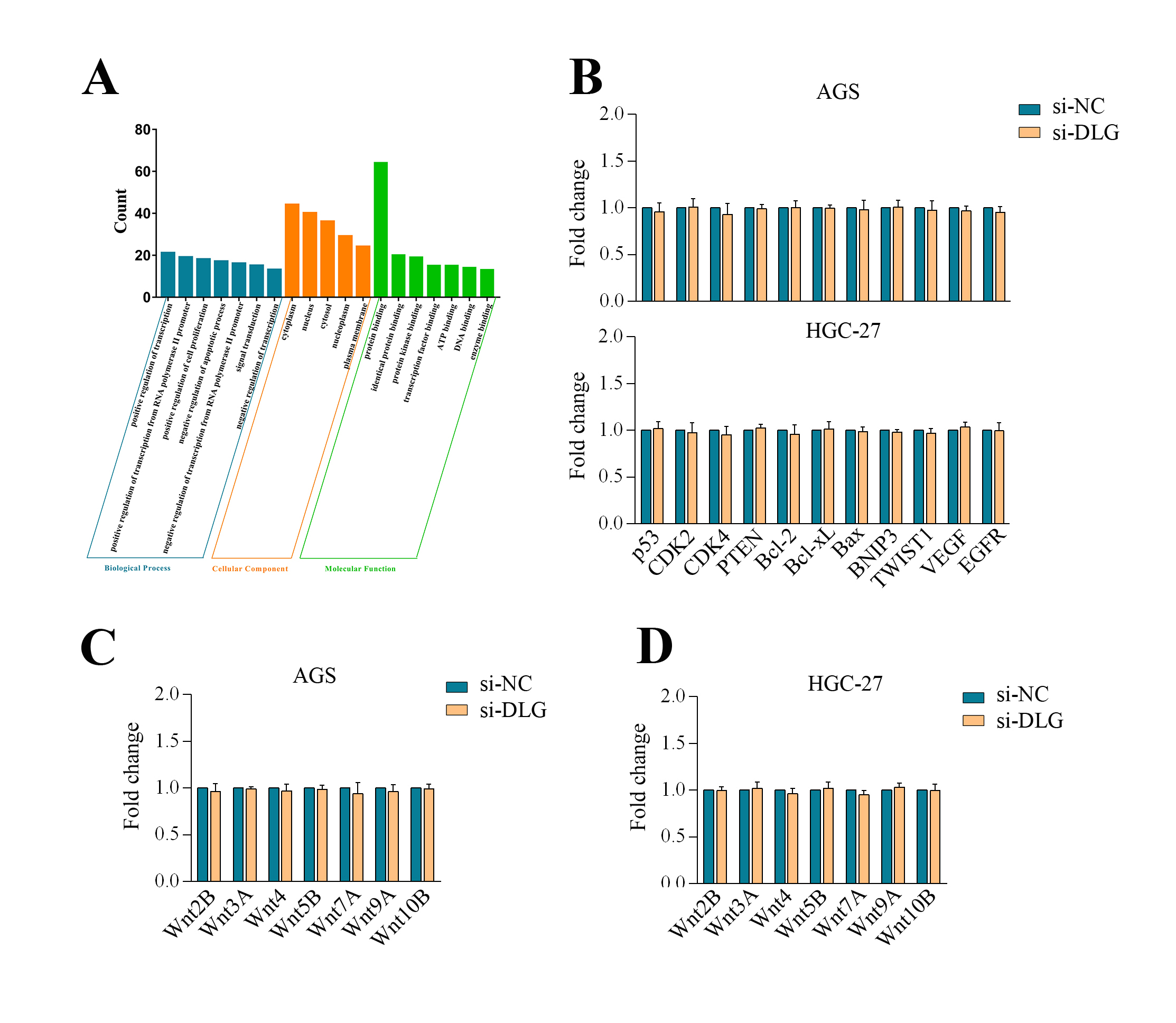

Supplement: Supplementary file 2 — Supplementary Figure 1 [file 41420_2021_649_MOESM2_ESM.jpg]

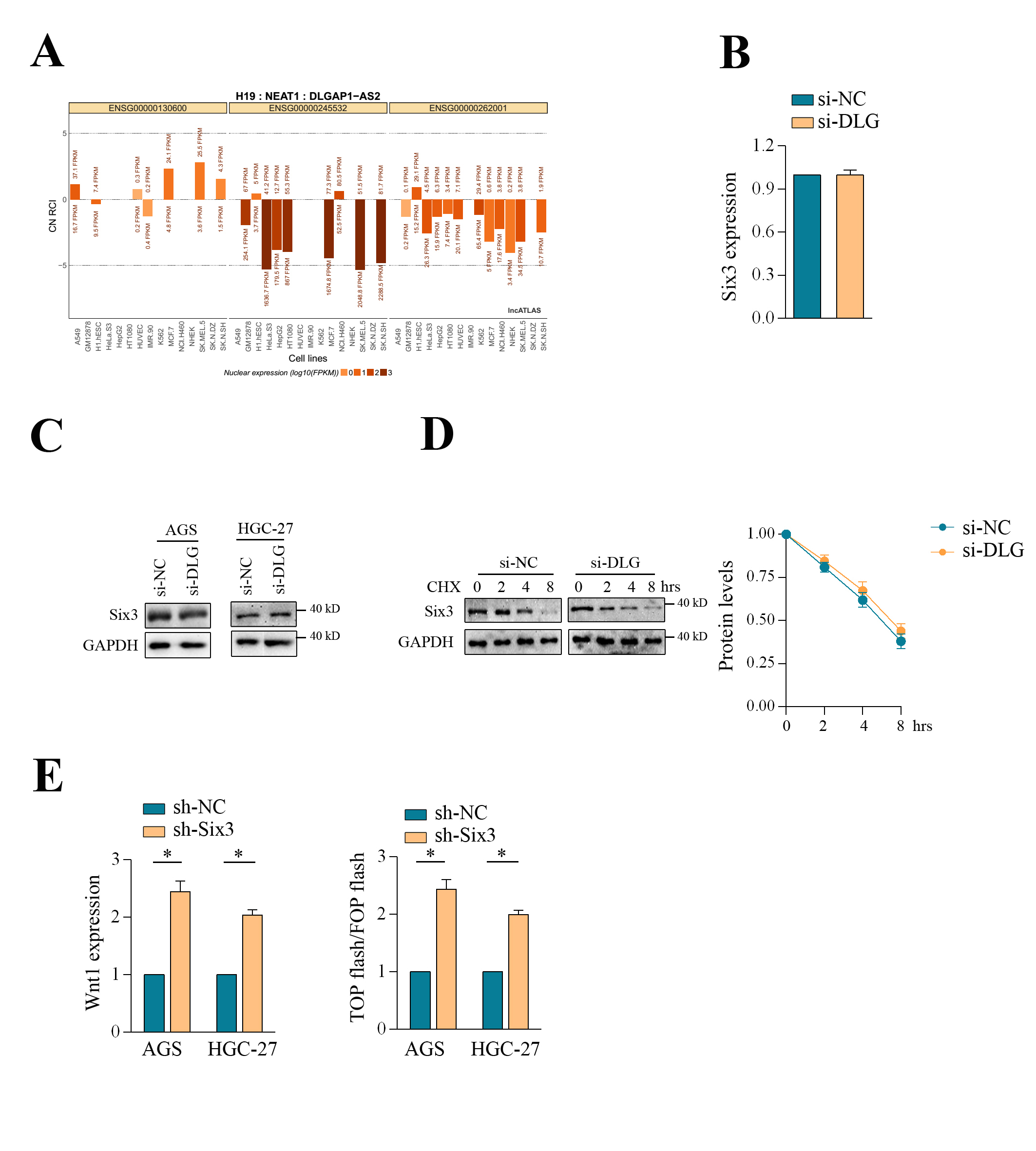

Supplement: Supplementary file 3 — Supplementary Figure 2 [file 41420_2021_649_MOESM3_ESM.jpg]

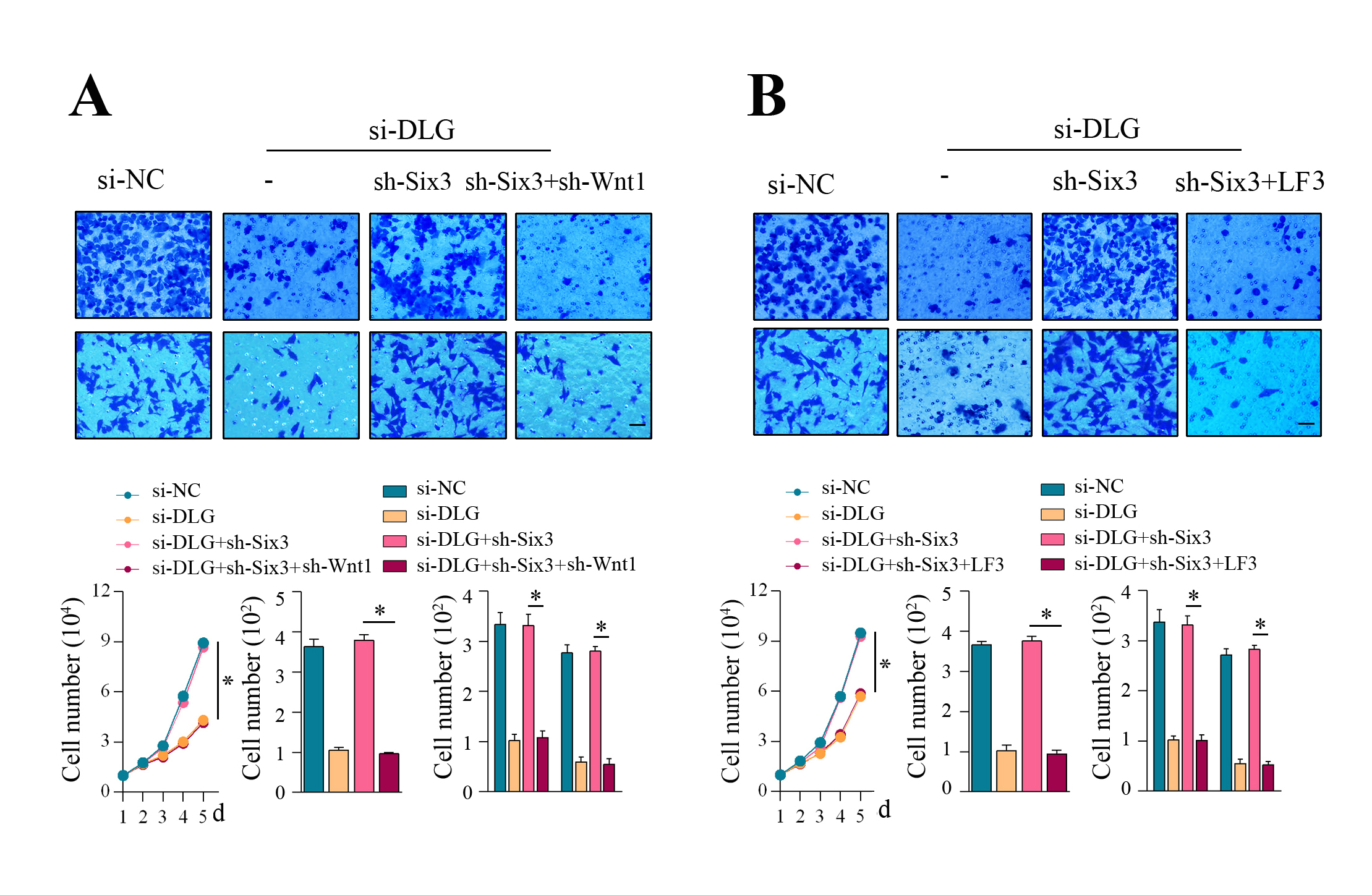

Supplement: Supplementary file 4 — Supplementary Figure 3 [file 41420_2021_649_MOESM4_ESM.jpg]
