# Supplementary material for: Long noncoding RNA DLGAP1-AS2 facilitates Wnt1 transcription through physically interacting with Six3 and drives the malignancy of gastric cancer
Source: Cell Death Discov. 2021 Sep 20;7:255. doi: 10.1038/s41420-021-00649-z (PMC8452735; doi:10.1038/s41420-021-00649-z)
Supplement: Supplementary file 5 — Supplementary Figure legend [file 41420_2021_649_MOESM5_ESM.docx]

**Supplementary Figure legend**

**Supplementary Figure 1 Screening of the possible targets downstream of DLGAP1-AS2.** (A) GO-term analysis of the co-expressed genes of DLGAP1-AS2 from TCGA STAD dataset. (B) RT-qPCR assays of a panel of tumor-associated genes involving in cell proliferation, apoptosis, EMT, and angiogenesis upon DLGAP1-AS2 silencing. (C) & (D) RT-qPCR analysis of the main family members of Wnt upon DLGAP1-AS2 silencing in AGS and HGC-27 cells.

**Supplementary Figure 2 Six3 expression level was unaffected upon DLGAP1-AS2 depletion.** (A) Prediction of subcellular distribution of DLGAP1-AS2 by the lncATLAS website tool. (B) & (C) The mRNA and protein expression of Six3 in GC cells upon DLGAP1-AS2 knockdown. (D) The stability of Six3 in DLGAP1-AS2 knockdown AGS cells after treatment with CHX (30 μg/mL). (E) The Wnt1 expression and TCF/LEF activity upon DLGAP1-AS2 knockdown. **P* < 0.05.

**Supplementary Figure 3 Inhibition of Wnt1 reversed the malignant phenotypes of HGC-27 cells driven by Six3 depletion.** (A) Colony formation assays, migration assays, invasion assays, and cell proliferation assays in HGC-27 cells upon transfection of si-NC, si-DLGAP1-AS2, si-DLGAP1-AS2+sh-Six3, or si-DLGAP1-AS2+sh-Six3+sh-Wnt1. (B) Colony formation assays, migration assays, invasion assays, and cell proliferation assays in HGC-27 cells upon transfection/treatment with si-NC, si-DLGAP1-AS2, si-DLGAP1-AS2+sh-Six3, or si-DLGAP1-AS2+sh-Six3+LF3. Data shown are representative of three independent experiments. Scale bar: 20 μm. **P* < 0.05.

**Supplementary Table 1 Primer or silencing sequences for the indicated genes.**
